# A Genetically Encoded Tool Kit for Manipulating and Monitoring Membrane Phosphatidylinositol 4,5-Bisphosphate in Intact Cells

**DOI:** 10.1371/journal.pone.0020855

**Published:** 2011-06-09

**Authors:** Fabian Hertel, Agathe Switalski, Elisa Mintert-Jancke, Katharina Karavassilidou, Kirsten Bender, Lutz Pott, Marie-Cécile Kienitz

**Affiliations:** Institute of Physiology, Ruhr-University Bochum, Bochum, Germany; University of Oldenburg, Germany

## Abstract

**Background:**

Most ion channels are regulated by phosphatidylinositol 4,5-bisphosphate (PtdIns(4,5)P_2_) in the cell membrane by diverse mechanisms. Important molecular tools to study ion channel regulation by PtdIns(4,5)P_2_ in living cells have been developed in the past. These include fluorescent PH-domains as sensors for Förster resonance energy transfer (FRET), to monitor changes in plasma membrane_._ For controlled and reversible depletion of PtdIns(4,5)P_2_, voltage-sensing phosphoinositide phosphatases (VSD) have been demonstrated as a superior tool, since they are independent of cellular signaling pathways. Combining these methods in intact cells requires multiple transfections. We used self-cleaving viral 2A-peptide sequences for adenovirus driven expression of the PH-domain of phospholipase-Cδ1 (PLCδ1) fused to ECFP and EYFP respectively and *Ciona intestinalis* VSP (Ci-VSP), from a single open reading frame (ORF) in adult rat cardiac myocytes.

**Methods and Results:**

Expression and correct targeting of ECFP-PH-PLCδ1_,_ EYFP-PH-PLCδ1, and Ci-VSP from a single tricistronic vector containing 2A-peptide sequences first was demonstrated in HEK293 cells by voltage-controlled FRET measurements and Western blotting. Adult rat cardiac myocytes expressed Ci-VSP and the two fluorescent PH-domains within 4 days after gene transfer using the vector integrated into an adenoviral construct. Activation of Ci-VSP by depolarization resulted in rapid changes in FRET ratio indicating depletion of PtdIns(4,5)P_2_ in the plasma membrane. This was paralleled by inhibition of endogenous G protein activated K^+^ (GIRK) current. By comparing changes in FRET and current, a component of GIRK inhibition by adrenergic receptors unrelated to depletion of PtdIns(4,5)P_2_ was identified.

**Conclusions:**

Expression of a FRET sensor pair and Ci-VSP from a single ORF provides a useful approach to study regulation of ion channels by phosphoinositides in cell lines and transfection-resistant postmitotic cells. Generally, adenoviral constructs containing self-cleaving 2A-peptide sequences are highly suited for simultaneous transfer of multiple genes in adult cardiac myocytes.

## Introduction

Phosphoinositides represent key molecules in numerous aspects of cellular signaling, including regulation of vesicular trafficking, cytoskeletal organization, cell cycle progression, and function of membrane proteins [1]. A large variety of ion channels and transporters are regulated by the abundance of phosphatitylinositol-4,5-bisphosphate (PtdIns(4,5)P_2_) in the inner leaflet of the plasma membrane [2]–[6]. PtdIns(4,5)P_2_ is the substrate of phospholipase C (PLC) isoforms and PI-3 kinases (PI3-K) and thus is the precursor of the second messengers IP_3_, DAG, and PtdIns(3,4,5)P_3_ [1]. PLC and PI3-K pathways in turn are regulated by cell type specific sets of heptahelical and tyrosine-kinase receptors. Moreover, the equlilibria between different phosphoinositides in different membrane compartments, including PtdIns(4,5)P_2_ in the plasma membrane, are controlled by activities of an array of lipid kinases and phosphatases and by phosphoinositide transfer proteins [1], [7], [8].

Research on PtdIns(4,5)P_2_-dependent signaling in the past has been furthered by methods for monitoring changes in PtdIns(4,5)P_2_ concentration using fluorescent PH-domains, preferentially as FRET pairs [9], [10], and by methods for controlled depletion of PtdIns(4,5)P_2_ either via native or expressed PLC-coupled receptors or techniques that are independent of cellular signaling pathways [11]–[16]. The most straightforward approach is based on the discovery of voltage-sensitive phosphatases (VSPs), initially cloned from the tunicate *Ciona intestinalis* (Ci-VSP, [17], [18]). These proteins consist of a voltage sensor domain with homology to subunits of voltage-activated K^+^ (Kv) channels and a phosphatase domain sharing homology to the phospholipid-phosphatase PTEN. VSPs specifically dephosphorylate PtdIns(3,4,5)P_3_ and PtdIns(4,5)P_2_ [18], [19]. Their phosphatase activity can be rapidly switched on and off in a graded manner by depolarization/repolarization [17].

Taking advantage of these experimental tools for investigating regulation of ion channels by PtdIns(4,5)P_2_ in mammalian cell lines requires co-expression of the VSP, two FRET sensors plus the channel or its subunits respectively. Feasibility of this approach in a mammalian cell line has been demonstrated recently in seminal studies analyzing PtdIns(4,5)P_2_ regulation of a voltage-activated K^+^-channel and L-type-Ca^2+^ channels by PtdIns(4,5)P_2_ [20], [21]. It should be noted, however, that the outcome of multiple transfections is difficult to control. Thus far these tools have not been applied to transfection-resistant primary cells, such as adult cardiac myocytes, for analyzing PtdIns(4,5)P_2_-dependent regulation of endogenous channels in their native signaling environment.

In the present study we generated a tricistronic shuttle vector containing the cDNAs for Ci-VSP and two fluorescent PH-domains (ECFP-PH-PLCδ1 and EYFP-PH-PLCδ1) concatenated through viral 2A-peptide sequences to yield a single open reading frame. The 2A-sequences result in co translational dissociation of the polyprotein while allowing translation to continue [22], [23]. Apart from minimizing the number of co-transfections, a major advantage of this approach results from the expression of the encoded proteins at comparable levels [24].

Expression and functionality of the two fluorescent PH-domains and the voltage-activated phosphatase, expressed from the single ORF is first verified in a standard mammalian cell line (HEK293). The molecular tool was then adapted for transfection resistant primary cells by construction of an adenovirus [25]. In simultaneous measurements of FRET and endogenous GIRK channel current in adult rat atrial myocyes infected with the tricistronic adenoviral vector, we can demonstrate that voltage activation of Ci-VSP results in rapid and reversible inhibition of current, paralleled by a transient reduction in FRET, which perfectly matches the time course of GIRK inhibition. We can demonstrate that activation of PLC by endogenous α_1a_ receptors results in inhibition GIRK channel current by PtdIns(4,5)P_2_ depletion and a mechanism that is independent thereof.

PH-domains of PLCδ1 bind PtdIns(4,5)P_2_ and G_βγ_. Their expression moderately slows replenishment of PtdIns(4,5)P_2_ after Ci-VSP-induced depletion but neither affects activation of GIRK channels by G_βγ_ nor PtdIns(4,5)P_2_ abundance required for GIRK channel function. We believe that our approach represents a versatile and powerful laboratory tool, facilitating investigations of regulation of membrane proteins by PtdIns(4,5)P_2_ but also of control and homoeostasis of levels of PtdIns(4,5)P_2_.

## Results

### Expression of two functional fluorescent PH-domains from a single ORF

**Figure 1:**
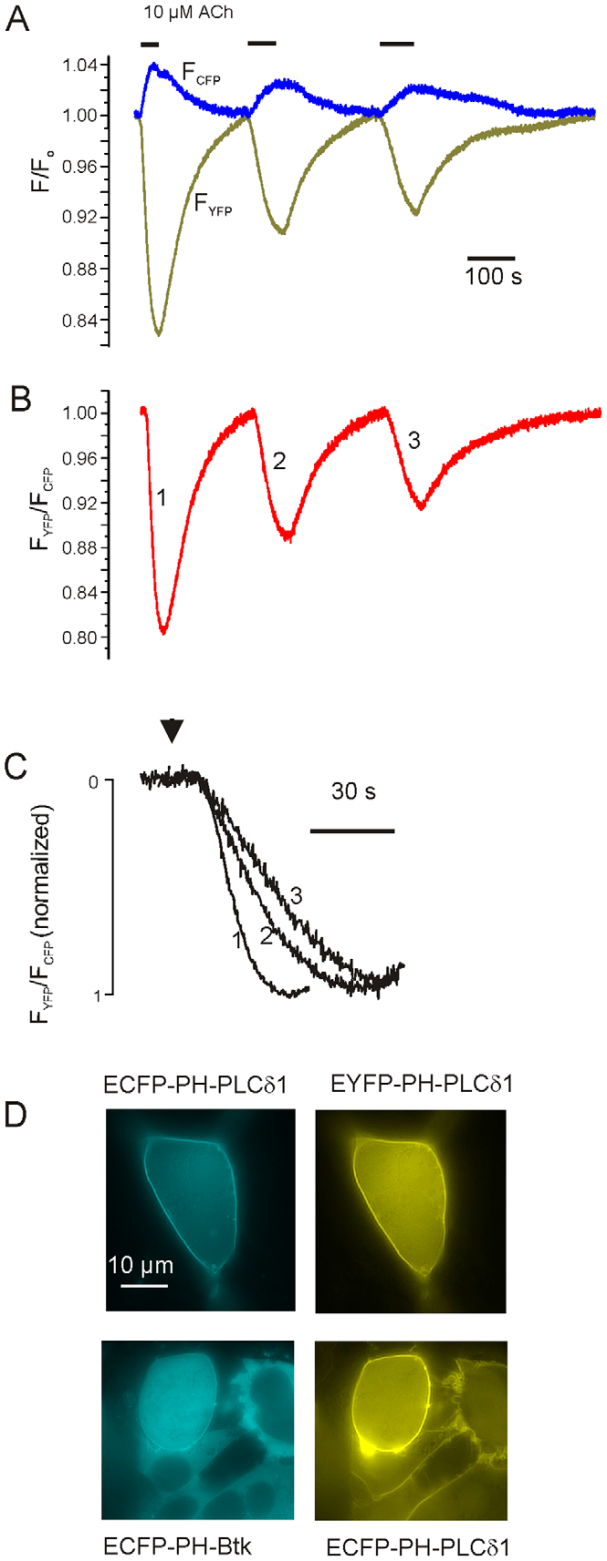
Functional expression of singular fluorescent proteins from 2A-peptide vectors. (A–C) Measurement of CFP and YFP fluorescence from a HEK293 cell cotransfected with pShuttle-EYFP-PH-PLCδ1P2A-ECFP-PH-PLCδ1 and a vector encoding for a muscarinic M_3_-receptor. (A) Representative recording of changes in fluorescence evoked by repeated exposures to Acetylcholine (ACh, 10 µM). (B) Corrected FRET ratio calculated from (A). (C) Rise times of changes in FRET from B on expanded time scale. D. Representative wide-field fluorescence images of localization of fluorescent PH-domains expressed from the tricistronic 2A-peptide vector pShuttle-PIP_2_-tool (top) in HEK-293 cells. In the bottom pair of images the sequence encoding for ECFP-PH-PLCδ1 was replaced by a sequence for ECFP-Btk-PH.

In a first step we verified functionality of the two fluorescent PH-domains expressed from the bicistronic vector pShuttle-EYFP-PH-PLCδ1P2A-ECFP-PH-PLCδ1 in HEK293 cells. For activation of PLC the cells were co-transfected with a pcDNA3 plasmid encoding for a PLC-coupled muscarinic M_3_ receptor. As shown in a representative time-resolved recording of single cell ECFP/EYFP fluorescence (Figure 1 A), exposure to acetylcholine resulted in an increase in ECFP fluorescence and a concomitant reduction in EYFP intensity, i.e. a decrease in FRET ratio (B), analogous to published data using transfection with conventional plasmids [26]. Though the muscarinic effect was completely reversible upon washout, the amplitude was reduced in consecutive responses. In parallel the rate of rise, which is governed by activity of PLC, was reduced (C), suggesting substantial desensitization of the receptor by the first exposure to ACh. This measurement, which is qualitatively representative of 6 cells identified as transfection-positive by EYFP-fluorescence, clearly demonstrates that the two PH-domains are expressed as singular proteins from the bicistronic 2A-peptide vector.

We next studied if the three proteins encoded by the tricistronic construct (pShuttle-PIP_2_-tool) are expressed, correctly processed and targeted to the plasma membrane, by transfecting HEK293 cells. As illustrated in Figure 1D, fluorescence of ECFP and EYFP showed predominant surface membrane localization, confirming expression of both PH-domains downstream of Ci-VSP. To corroborate that fluorescence from ECFP and EYFP did not originate from uncleaved tandem proteins, we used a vector, in which the sequence of ECFP-PH-PLCδ1 was replaced by ECFP-PH-Btk. This domain does not bind PtdIns(4,5)P_2_ but PtdIns(3,4,5)P_3_, whose abundance in the plasma membrane is very low in the absence of an activator of PI3-kinase [27]. In cells transfected with this vector, ECFP fluorescence was diffusively distributed in the cytoplasm, while EYFP fluorescence was still predominantly localized to the plasma membrane. Localization of ECFP/EYFP fluorescence to different compartments unambiguously demonstrates expression of the two individual fluorescent proteins.

### Functional expression of Ci-VSP

**Figure 2:**
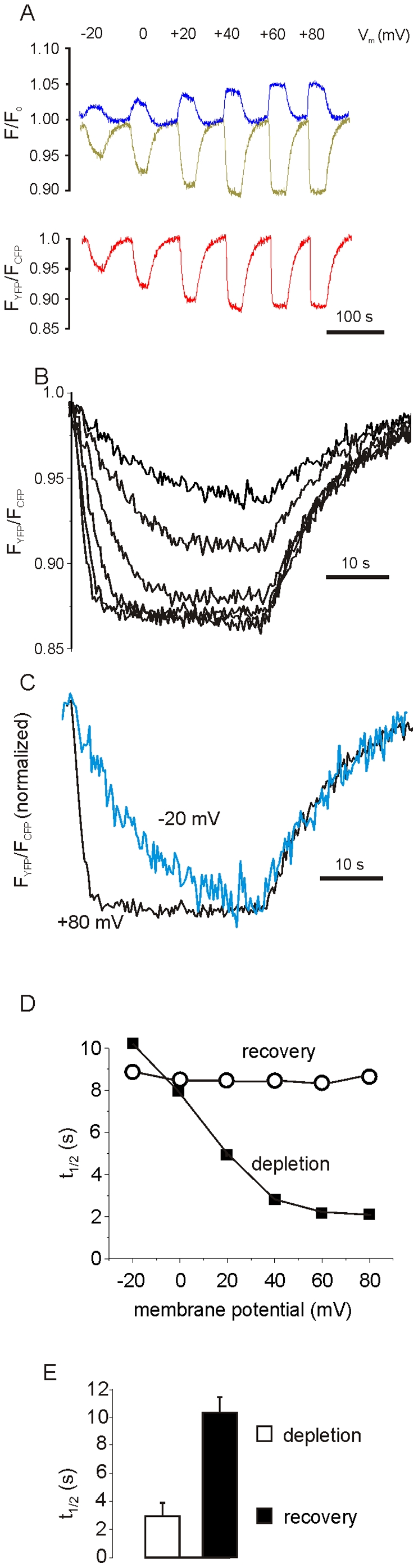
Continuous recording of ECFP and EYFP fluorescence from a single HEK293 cell transfected with pShuttle- PIP_2_-tool. The cell was voltage-clamped in whole cell mode. Fluorescence changes (A) were evoked by step depolarizations of 30 s in duration from −90 mV holding potential to the levels indicated. The resulting corrected fluorescence signals (F_EYFP_- yellow trace and F_ECFP_- blue trace) were normalized to values before agonist application (F*/*F_o_) or expressed as FRET ratio (F_EYFP_/F_ECFP_, red trace). (B) Superimposed changes in FRET ratio from A on expanded timescale. (C) Changes in FRET ratio evoked by voltage-steps to −20 mV (blue) and +80 mV (black) were superimposed and scaled to match the steady-state levels. (D) Plot of t_1/2_ for rise time (depletion) and recovery of FRET signals against membrane potential. (E) Mean values±SEM of half times of FRET signal indicating PtdIns(4,5)P_2_-depletion and recovery by voltage steps to +80 mV (n = 6).

The positive detection of the ECFP/EYFP-fused PH-domains, which are downstream of the Ci-VSP-T2A sequence, predicts that Ci-VSP was expressed, C-terminally fused with 20 residues from the T2A-peptide [28]. In the majority of cases, fusion of a protein with a 2A-peptide moiety does not compromise its function. However, examples of proteins that are functionally disturbed by the peptide residues have been published [24], [29]. As illustrated in figure 2 (A–D), in HEK293 cells transfected with pShuttle-PIP_2_-tool, graded changes in FRET ratio, indicating depletion of PtdIns(4,5)P_2_, were induced by depolarizing voltage steps. Both, the amplitude and the on-kinetics of the FRET-response were voltage-dependent and, in line with previous studies, saturated at membrane potentials positive to +60 mV [18]. The kinetics of recovery upon repolarization, which should be governed by replenishment of PtdIns(4,5)P_2_ via phosphatidylinositol-4-phosphate 5-kinase, was independent of the preceding depolarization (C, D). Both the kinetics of depletion at saturating voltage and the recovery showed little variation in different cells (E). In conclusion, these data demonstrate expression and correct processing of the three proteins encoded by the single ORF in a standard mammalian cell line.

**Figure 3:**
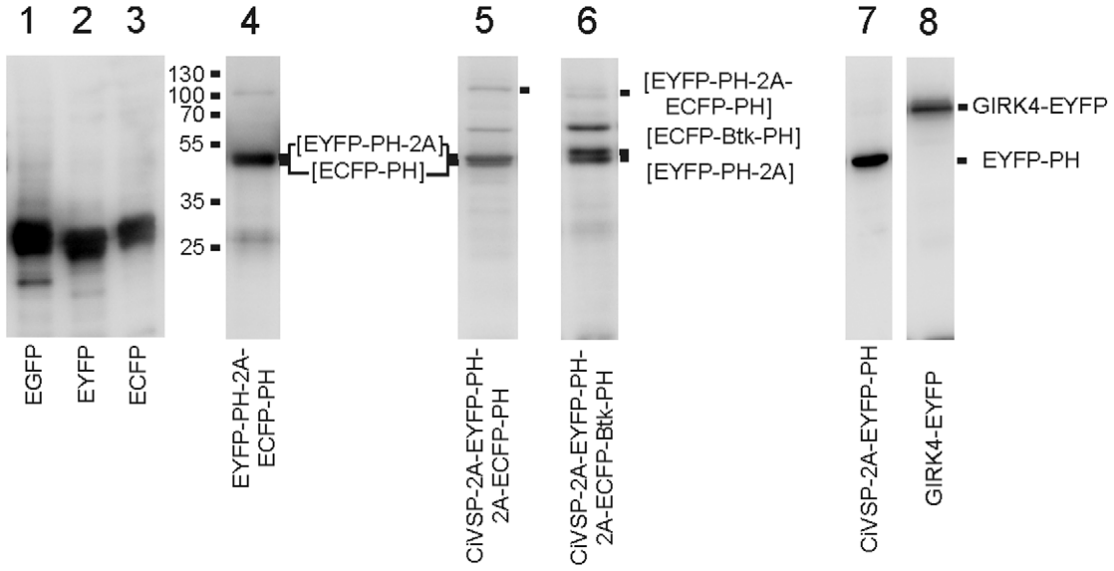
Western blot analyis of the expression of ECFP/EYFP-PH-PLCδ1 and polyproteins in transfected HEK293 cells (see text for details).

Positive detection of voltage-dependent changes of FRET ratio does not exclude that a fraction of the proteins escapes the self-cleaving mechanism. The relative amount of uncleaved products was estimated by Western blots using a polyclonal GFP antibody. As illustrated in Figure 3 (lanes 1–3) this antibody equally detects EXFP variants. In HEK293 cells transfected with pShuttle-EYFP-PH-PLCδ1P2A-ECFP-PH-PLCδ1 (lane 4) the amount of uncleaved protein was negligible in comparison with the two singular PH-domains. This pattern was similar in lysates from cells transfected with pShuttle-PIP_2_-tool (lane 5), or the analogous vector containing the sequence for Btk-PH (lane 6). For completeness, lane 7 demonstrates cleavage of Ci-VSP2A and EYFP-PH and detection of a genuine EYFP-fused membrane protein (GIRK4, lane 8). An unidentified XFP-positive band sometimes appeared at MW of about 60 KD (lanes 5, 6), which is unrelated to any of the fluorescent constructs. According to the manufacturer's information, this is an artefact produced by the antibody itself.

### Adenovirus-driven expression in adult atrial myocytes

**Figure 4:**
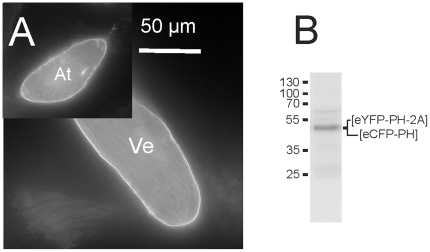
Fluorescent PH-PLCδ1 are expressed in adult cardiac myocytes after infection with Ad-PIP_2_-tool. Membrane localization of EYFP fluorescence in adult ventricular (Ve) and atrial (At) myocyte from heart of adult rat on day 4 after infection. Note that in line with previous studies, atrial myocytes show a tendency to round up within about two days *in vitro*, whereas ventricular myocytes under identical conditions retain their typical brick-shaped morphology for more than one week (B) Western blot from lysate of atrial myocytes 4 d after infection with Ad-PIP_2_-tool.

In order to make this molecular tool applicable to postmitotic transfection-resistant cells, the tricistronic pShuttle-CMV vector was used for recombination with the pAdEasy-1 backbone plasmid to generate a recombinant adenovirus (Ad-PIP_2_-tool). After infection with the virus, adult rat atrial and ventricular myocytes showed membrane-delimited fluorescence within ≥48 h (figure 4 A). In Western blots the singular fluorescent PH-domains were detected (B). Uncleaved protein was below detection limit. To demonstrate proof of principle and usefulness of this approach, we used endogenous GIRK current of atrial myocytes carried by tetrameric complexes of Kir3.1 and Kir3.4 [30]. This channel is opened by binding of G_βγ_ upon activation of muscarinic M_2_-receptors and other GPCRs that converge on pertussis toxin-sensitive G_i/o_. The observation that this process requires the binding of PtdIns(4,5)P_2_ represents a key finding in this field [3], [31].

**Figure 5:**
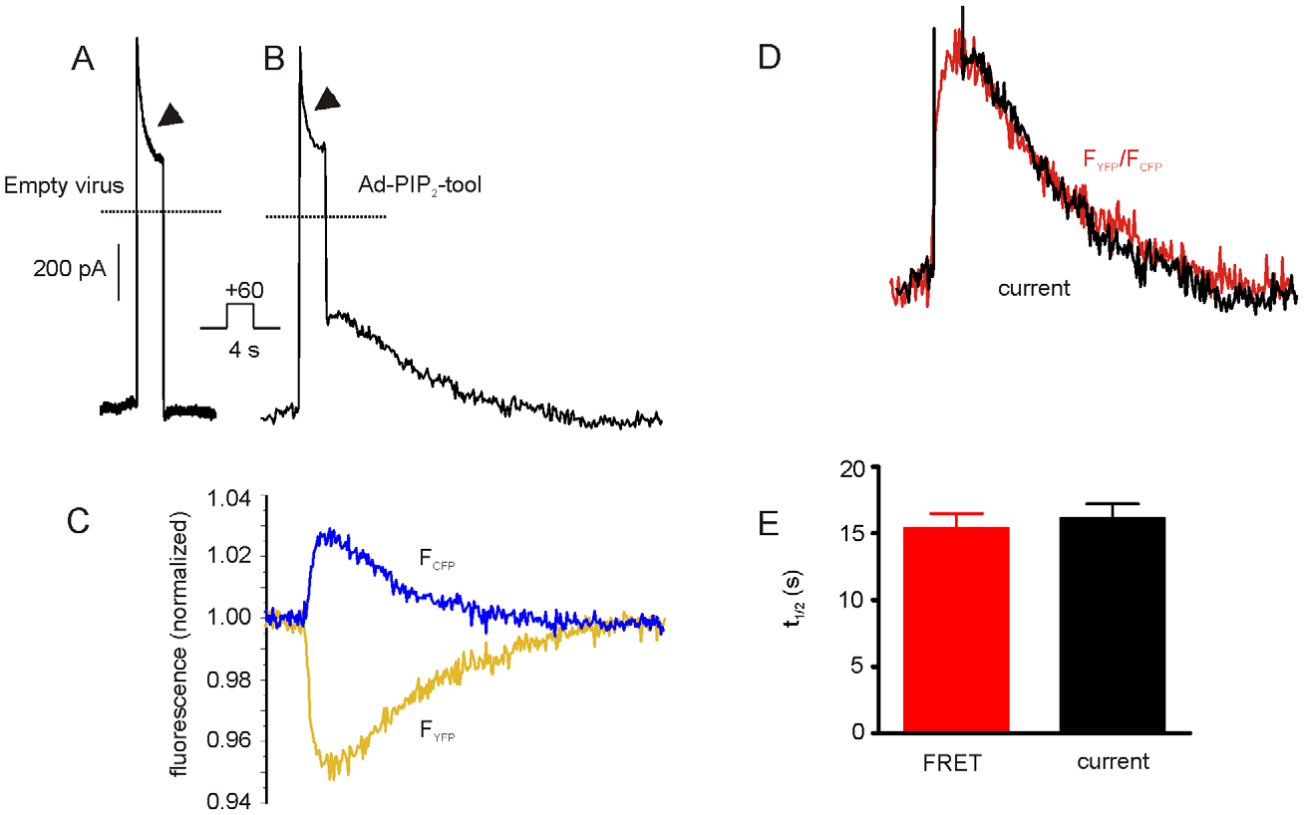
Inhibition of GIRK channel current and changes in EYFP/ECFP fluorescence induced by a step depolarization from −90 mV to +60 mV in an atrial myocyte infected with Ad-PIP_2_-tool. GIRK currents were stably activated using GTP-γ-S in the pipette filling solution as illustrated in Figure S2. Changes in membrane current recorded from myocytes infected with empty virus (A) or Ad-PIP_2_-tool (B). The arrowheads mark the current relaxation dominated by the inward-rectifying mechanism (see text for details). Dotted lines indicate zero current determined by 2 mM Ba^2+^, as in Figure S2. (C) Recordings of fluorescence simultaneous with current recording (trace B). (D) Superimposed traces representing FRET-ratio (from C) and recovery of current from A. The two traces were scaled to match their peaks. E. Comparison of half times of recovery (t_1/2_) of FRET and current (n = 40 myocytes).

**Figure 6:**
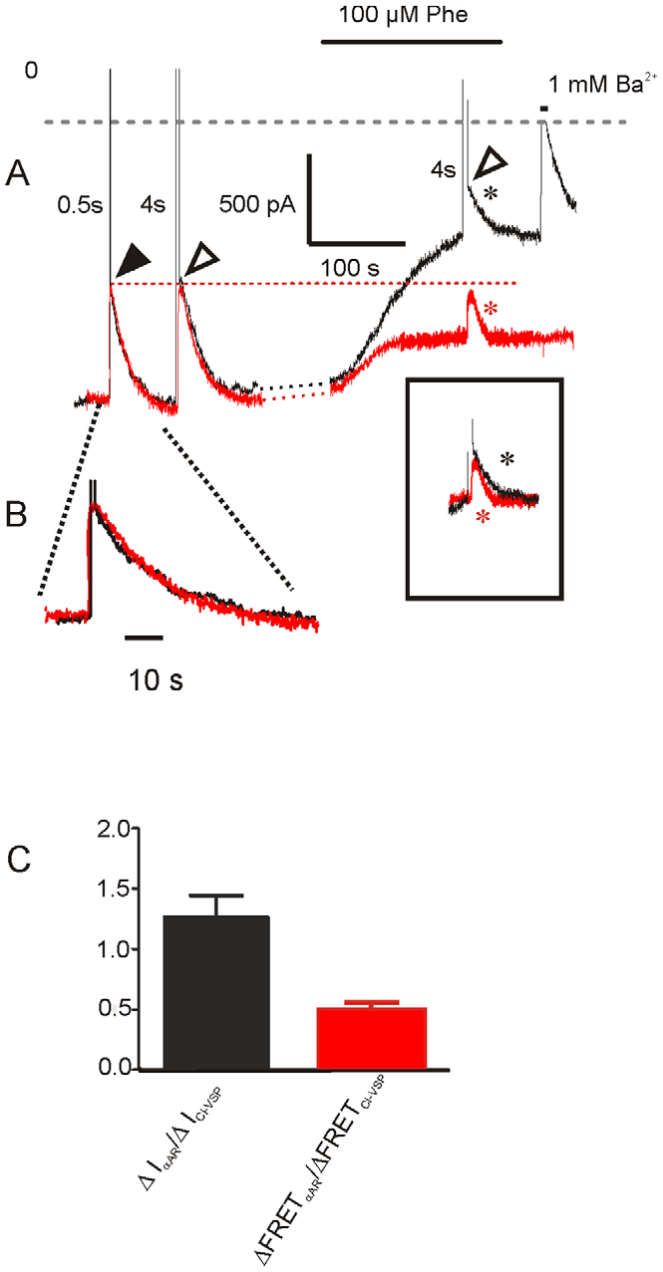
Differential changes in GTP-γS-activated GIRK current and EYFP/ECFP FRET ratio by voltage activation of Ci-VSP and α_1a_ adrenergic receptors using phenylephrine in a myocyte infected with Ad-PIP_2_-tool. (A) Continuous recording of membrane current (black trace) and FRET-ratio (red trace). An interruption of about 90 s in duration is denoted by the dotted lines. The arrowheads indicate responses to step depolarization to +60 mV of 0.5 s (black) and 4 s (open) in duration. The red and black trace were scaled to match the peak of the responses indicated by the closed arrowhead, resulting in superimposable time-courses (B); the dotted red line marks the current level at repolarization and the peaks of superimposed FRET transients. At the time indicated, the cell was superfused with Phe-containing solution. In the inset the two transients marked by the asterisks were superimposed. BaCl_2_ was applied as indicated to define total inward-rectifier current. (C) Ratios of current inhibition by α_1_-AR activation and voltage activation of Ci-VSP (ΔI_αAR_/Δ_ICi-VSP_, black column) and corresponding FRET signals (ΔFRET_αAR_/ΔFRET_Ci-VSP_, red column; n = 8).

In atrial myocytes GIRK channel current upon activation via the canonical muscarinic M_2_ receptor undergoes desensitization by various mechanisms [32]–[34]. To maintain a stable level of GIRK channel current, myocytes were loaded with GTP-γ-S (200 µM) by inclusion in the filling solution of the patch-clamp pipette, which results in a stable current level after brief exposure(s) to ACh, as illustrated in figure S2 (A). Because of the strong inward rectification of GIRK channels (figure S2 B) it is advantageous to record Kir3 channel currents in the inward direction, i.e. at membrane potentials negative to E_K_ (−50 mV in the present conditions). Moreover, a holding potential negative to −40 mV at the least, is important for preventing basal activation of Ci-VSP [35]. Figure 5 compares traces reflecting the change in membrane current caused by a step depolarization from −90 to +60 mV (4 s) recorded from a myocyte infected with an empty virus (A) or Ad-PIP_2_-tool (B). The depolarization resulted in an instantaneous outward current that rapidly declined also in native or mock-infected myocytes due to the block by intracellular polyamines and Mg^2+^ underlying inward rectification [36], [37]. The relaxation of outward current impedes identification of an effect of PtdIns(4,5)P_2_ depletion on outward GIRK current by voltage-activation of Ci-VSP. Upon repolarization the baseline current level is reached quasi instantaneously in native or mock-infected cells, due to the rapid kinetics of the unblock. The effect of Ci-VSP-induced depletion of PtdIns(4,5)P_2_ can be identified after repolarization as a reduction of inward current, which recovers with a half time of about 11 s in this cell. The changes in fluorescence of ECFP and EYFP recorded simultaneously with membrane current have been traced in panel C. The resulting change in FRET ratio, superimposed and scaled on the current trace (D), perfectly matches the recovery of current, which was a consistent finding (E), suggesting that PtdIns(4,5)P_2_-binding affinities of the PH-domains and Kir3-subunits are similar. As a rule, a depolarization to +60 mV of 4 s in duration was saturating in terms of both, inhibition of current and FRET signal (see also figure 6). Interestingly, inhibition was never complete but saturated at 73.5±6.7% (n = 40). The incomplete inhibition by saturating activation of Ci-VSP is in line with data obtained from *Xenopus* oocytes coexpressing Kir3.2 and Ci-VSP [35].

### A component of GIRK current inhibition unrelated to depletion of PtdIns(4,5)P_2_

Inhibition of GIRK current in atrial myocytes by stimulation of endogenous G_q_-coupled receptors such as α_1a_ adrenergic or endothelin-1 has been demonstrated in several publications. It is commonly accepted that this inhibition is primarily or entirely due to depletion of PtdIns(4,5)P_2_ [11], [38], [39]. On the other hand, in heterologous expression systems and in hippocampal neurons evidence for an additional inhibitory effect via PKC-mediated phosphorylation of Kir3 subunits has been provided. As underlying mechanism a reduction in PtdIns(4,5)P_2_-binding of the phosphorylated channel has been suggested [40]–[43]. Figure 6 illustrates a representative experiment, in which effects of signal-independent PtdIns(4,5)P_2_-depletion by Ci-VSP and activation of α_1_ receptor activation by phenylephrine (Phe, 100 µM) were compared. Analogous to Figure 5, the matched Ci-VSP-induced signals reflecting current inhibition and inverted change in FRET-ratio were perfectly superimposable (A, B). Moreover, in this cell changes in current and FRET were identical for voltage steps to +60 mV of 0.5 s and 4 s in duration, suggesting that these were saturating responses in terms of PtdIns(4,5)P_2_ depletion. Exposure to Phe resulted in a stronger reduction of current as compared to saturating activation of Ci-VSP, whereas the change in FRET was substantially smaller. As summarized in Figure 6 C, the mean inhibition of current was about 30% larger by Phe as compared to Ci-VSP, whereas the change in FRET ratio was 50% less. Voltage activation of Ci-VSP in the presence of Phe caused additional inhibition of current, paralleled by a superimposable change in the FRET trace, ruling out that the loss in correlation between FRET and current upon α-adrenergic stimulation can be accounted for by a reduction in sensitivity of the FRET sensors. This clearly suggests that a sizable fraction of GIRK current inhibition by activation of the G_q_ /PLC pathway does not directly reflect depletion of PtdIns(4,5)P_2_.

### Expression of PH-domains slows replenishment of PtdIns(4,5)P_2_

**Figure 7:**
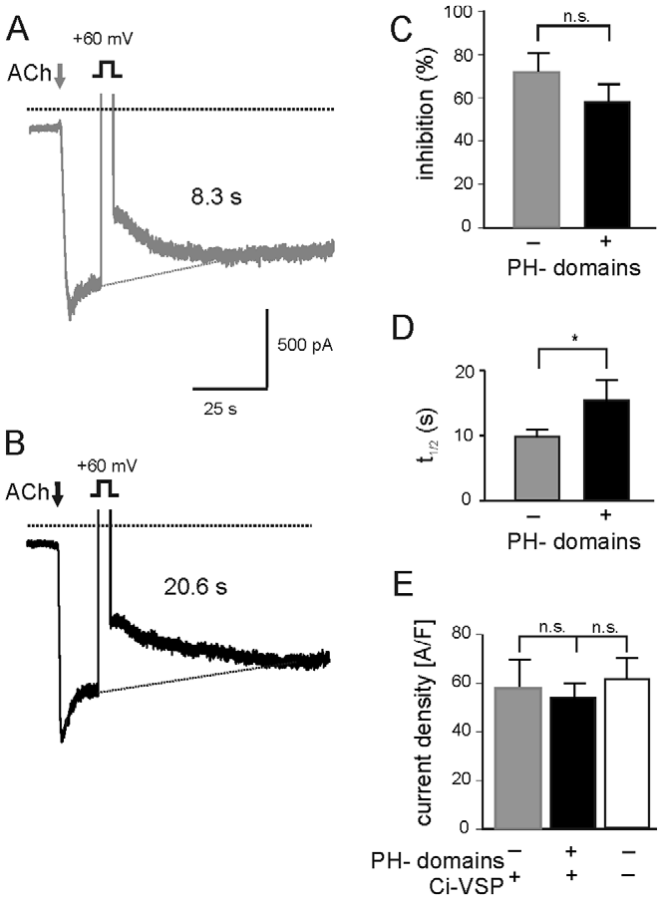
Expression of PH-domains affects recovery of GIRK current after Ci-VSP-induced depletion of PtdIns(4,5)P_2_. In order to allow for normal G protein cycling, pipette filling solutions die not contain GTP-γ-S but GTP (20 µM). Representative recordings of ACh-activated current from myocytes infected with Ad-Ci-VSP (A) or Ad-PIP_2_-tool (B). At the time indicated a depolarization to +60 mV of 4 s in duration was applied (outward current blanked out). Numbers denote half times of recovery to baseline. Baseline was extrapolated as indicated by dotted lines because of desensitization. (C) Summarized percentage of inhibition of current by Ci-VSP using a step depolarization to +60 mV of 4 s in duration (D) Summarized half times of current recovery. (E) Summarized densities of peak currents. (n = 18 without PH-domains; n = 22 with PH-domains for C, D, and E; n = 40 for the white column in panel E).

Fluorescent PH-PLCδ1 binds PtdIns(4,5)P_2_ with a K_D_ in the order of 1 µM. Their expression inevitably causes a reduction in the effective membrane concentration of the phosphoinositide. Depending on the expression level, this might exert a tonic inhibitory effect on PtdIns(4,5)P_2_-sensitive proteins and, by competition with PIP-kinase(s), could affect kinetics of replenishment of PtdIns(4,5)P_2_ [44], either by a direct buffering effect or by a compensatory increase in expression of PIP-kinases [45], [46]. Moreover, PH-PLC domains can bind βγ subunits of heterotrimeric G proteins and therefore can interfere with signaling via G_βγ_ [47]. In order to test, to what extent these properties might be limiting applicability of the FRET sensors, we measured GIRK current in atrial myocytes expressing either Ci-VSP alone or in combination with the two fluorescent PH-domains. Current densities and rates of recovery of current after voltage-activation of Ci-VSP were compared. Figure 7 shows representative current traces recorded from myocytes infected either with Ad-Ci-VSP (A) or Ad-PIP_2_-tool (B). GIRK current in these experiments was activated by 2 µM ACh with GTP instead of GTP-γ-S in the pipette solution to maintain normal G protein cycling. Step depolarizations to +60 mV of 4 s were used to activate Ci-VSP, resulting in comparable inhibition of current by 45% and 40% respectively in these individual cells. The decrease in current by activation of Ci-VSP using this voltage step was not different with and without co-expression of the PH-domains (Figure 7 C), suggesting comparable levels of expression from the two different adenoviral vectors. Half times of recovery were 8.3 s in the cell infected with Ci-VSP only and 13.5 s with co-expressed PH-domains. This difference is significant (figure 7 D), i.e. in contrast to previous studies using a mammalian cell line, in cardiac myocytes resynthesis of PtdIns(4,5)P_2_ appears to be moderately slowed by the expressed PH domains. Density of ACh-activated current was neither reduced by expression of PLC co-expressed with Ci-VSP nor by Ci-VSP alone (figure 7 E) suggesting that in the present conditions neither signaling via βγ complexes released from G_i/o_ is affected nor is the basal concentration of PtdIns(4,5)P_2_ in the inner leaflet of the plasma membrane reduced by expression of the PH-domain or Ci-VSP.

## Discussion

Since the first publication demonstrating sensitivity of an ion channel to PtdIns(4,5)P_2_ [2], this has been confirmed for a number of around 40 ion channel species of diverse families [48]. Sensitivity of many ion channels to PtdIns(4,5)P_2_ has been localized to the pore-forming protein complex, but other mechanisms, such as interaction of PtdIns(4,5)P_2_ with a regulatory β-subunit have been identified [49]. The majority of PtdIns(4,5)P_2_-sensitive channels and transporters are activated or kept in functional state by PtdIns(4,5)P_2_; however, inhibition of several ion channel species by PtdIns(4,5)P_2_ has been described. Interestingly this includes native cardiac I_Ks_ constituted by KCNQ1/KCNE1(e.g. [50], [51]. The relevance of regulation of ion channel functions by PtdIns(4,5)P_2_ in a physiological context is largely unknown, with few exceptions, such as the neuronal KCNQ2/3 (M-current; e.g, [5], [52], [53]), and the atrial GIRK current used as paradigmatic PtdIns(4,5)P_2_ –sensitive species in the present study (e.g. [54]). For investigating normal and disturbed regulation of ion channels by PtdIns(4,5)P_2_, expression of molecular tools to manipulate and monitor PtdIns(4,5)P_2_ levels with simultaneous measurement of channel function in their native signaling environment are desirable.

For manipulation of PtdIns(4,5)P_2_ endogenous or expressed GPCRs that activate PLCs have been used in various studies in the past. This has furthered our knowledge on regulation of ion channels-inward rectifiers and KCNQ channels in particular–by this ubiquitous pathway (e.g. [53], [55]–[57]. However, PLC activation results in cellular signals downstream of IP_3_/Ca^2+^ and PKC, and various ion channels, including Kir3, have been shown to be modulated by PKC phosphorylation or by Ca^2+^ [43], [58]. A clear identification of effects specifically related to depletion of PtdIns(4,5)P_2_ is difficult for channels that are targets of these pathways. Research in this field has been promoted by the development of PH-domain-derived fluorescent PtdIns(4,5)P_2_ sensors [9], [10] and by experimental tools for depleting membrane PtdIns(4,5)P_2_ without activation of signaling pathways. Two laboratories introduced an elegant approach for selectively depleting PtdIns(4,5)P_2_ by rapamycin-inducible membrane recruitment of an inositol-phosphatase [14], [15]. Depletion of PtdIns(4,5)P_2_ using this technique is rapid and selective but lacks reversibility. The remarkable potential of voltage-activated phosphatases, which can be rapidly and reversibly controlled by membrane potential on a sub-second time-scale in single cells, has been demonstrated in a number of recent studies using *Xenopus* oocytes or mammalian cell lines [18], [19], [35]. To date this tool had not been used to study regulation of an ion channel species in a native cell background.

Standard approaches to achieve co-expresssion of proteins are transfection with several conventional or viral vectors, containing IRES sequences or multiple promoters. These strategies have been used successfully in many studies. However, the number of proteins that can be reliably expressed is limited and difficult to control. In the present work we used a molecular strategy to express two PtdIns(4,5)P_2_-sensitive fluorescent sensor proteins and a voltage-activated phosphatase from a single ORF in terminally differentiated cardiac myocytes. The 2A-peptide approach has been used in the past e.g. for reconstituting protein complexes such as the T-cell CD3 complex [59] or to express four transcription factors required for inducing IPS cells [60]. Proof of principle of the 2A-peptide approach for expression of three different proteins has been demonstrated recently in primary cultured neurons [61], but surprisingly, employment of this useful strategy for manipulating cardiac myocytes to date is lacking. Since 2A-peptide sequences cause co-translational cleavage of the individual proteins by driving a termination reaction at the ribosome [28], the expression levels of the individual proteins are comparable, if not identical [60]. This represents a particularly advantageous condition for measuring FRET between two fluorescent proteins, resulting in an excellent signal-to-noise ratio of the fluorescence signals. Like in any reporter assay that is based on binding of the molecule to be measured, the expressed PH-domains compete with other PtdIns(4,5)P_2_ binding proteins, such as ion channel complexes. This “buffering” effect could affect basal PtdIns(4,5)P_2_ levels as well as rates of recovery subsequent to depletion. Using endogenous GIRK current as sensor we can demonstrate that expression of the PH-domains moderately slows recovery of current subsequent to CI-VSP-induced depletion of PtdIns(4,5)P_2_, presumably reflecting a buffering effect. Current density of ACh-activated GIRK current was unaffected by expression of PH-domains. This might suggest that the reduction of the abundance of PtdIns(4,5)P_2_ by expression of the PH-domains is compensated by an increase in expression of the PtdIns(4,5)P_2_-generating kinases, as has been suggested in ref. 46. Since GIRK channels are sensitive detectors of G_βγ_, this also excludes significant sequestration of the βγ-complexes involved in channel activation.

Our study shows the utility of using multicistronic vectors for simultaneous expression of the present state-of-the art molecular tools for manipulating and measuring the levels of plasma membrane-associated PtdIns(4,5)P_2_. This approach facilitates investigating the PtdIns(4,5)P_2_-dependence of ion channels with cell-type specific composition of pore-forming and ancillary subunits in their native signaling environments.

The modular nature of the tricistronic vector allows for easy replacement of cDNAs encoding for different sensor constructs (see figure 1 D) but also for VSP-species, such as Dr-VSP cloned from *Dario*, which have a voltage-range of activation better suited for investigating voltage-dependent ion channels [21], [46], [62]. VSP constructs with altered substrate specificity and suitable sensors for studying subcellular dynamics of other inositolphosphates are further interesting options. Moreover, the universal CMV promoter used in the present study can be replaced by myocyte-specific or inducible promoters for *in vitro* or *in vivo* studies.

## Materials and Methods

### Molecular Biology

The cDNAs of the PtdIns(4,5)P_2_ binding PH domain of phosfpholipase C_δ1_ (PLC_δ1_, amino acids 1-153) and the PtdIns(3,4,5)P_3_ binding PH domain of Bruton's tyrosine kinase (Btk, amino acids 1-177) were amplified by RT-PCR from rat brain total RNA with the following primer pairs:

**PLCδ1** **-** 5′-GGATCCATGGACTCGGGTAGGGACTT-3′ and

5′-GCGGCCGCTTAGAATTCATCAGCCTTTCGCAAGCA-3′;

**Btk -** 5′-GGATCCATGGCTGCTGTGATACTGGA-3′ and

5′-GCGGCCGCTTAGAATTCTTTTAAGCTTCCATTCCTGTTCT-3′

The underlined triplets serve as a variable stop codon. Recombinant fluorescent probes were generated by inserting the cDNA of the PH domains in frame behind ECFP or EYFP in pcDNA 3 (+) (Invitrogen) using a BamHI restriction site as linker.

A multicistronic 2A peptide based vector for expression of Ci-VSP, EYFP-PH-PLCδ1, and ECFP-PH-PLCδ1 was obtained by linking the individual cDNAs with viral 2A oligopeptide sequences as outlined in figure S1. DNA sequences of 2A peptides, derived from insect virus *Thosea asigna* (T2A) and porcine Teschovirus-1 (P2A), and appropriate restriction sites for recombination were attached to the cDNAs of Ci-VSP (kindly provided by Y. Okamura, Osaka, Japan) without stop codon and ECFP-PH-PLCδ1 in a second amplification reaction by PCR using the following primer pairs:

**Ci-VSP-** 5′-AAGCTTAGATCTGATATCGCCACCATGGAGGGATTCGACGGTT-3′ and

5′-AAGCTTAAGTGGGCCGGGATTTTCCTCCACGTCCCCGCATGTTAGAAG

ACTTCCCCTGCCCTCGCCGGAGCCTCCGGAAATGTCTTCAGCATCTGAAAACGT

AAG-3′;

**ECFP-PH-** 5′-GAATTCACCGGTGGCAGCGGCGCCACAAACTTCTCTCTGCTAAA

GCAAGCAGGTGATGTTGAAGAAAACCCCGGGCCTTGGCGCGCCATGGTG

AGCAAGGGCGAGGA-3′ and

5′-GCGGCCGCGTTAACTTACGTACGATCAGCCTTTCGCAAGCAGG-3′.

(The underlined sequences encode for T2A and P2A). All PCR products were cloned into pCR2.1 Topo TA Cloning vector (Invitrogen) and verified by sequencing. The extended cDNAs of ECFP-PH-PLCδ1, and EYFP-PH-PLCδ1 were subcloned in series in pShuttle-CMV to generate either pShuttle-EYFP-PH-PLCδ1P2A-ECFP-PH-PLCδ1 or in combination with Ci-VSP2A (pShuttle-Ci-VSPT2A-EYFP-PH-PLCδ1P2A-ECFP-PH-PLCδ1, termed pShuttle- PIP_2_-tool for convenience) Using analogous procedures, a vector was constructed, in which ECFP-PH-PLCδ1 was exchanged for ECFP-PH-Btk (Brutońs tyrosine kinase) to generate pShuttle-Ci-VSPT2A-EYFP-PH-PLCδ1P2A-ECFP-PH-Btk. For expression of Ci-VSP without PH-domains the cDNA was subcloned in pAdtrack-CMV, which contains a GFP sequence to generate pAd-Ci-VSP.

### Construction of adenovirus

The pAd-Easy1 plasmid encoding for the adenovirus type 5, pAd-Track-CMV, and pShuttle-CMV were kindly provided by Dr B. Vogelstein (Johns Hopkins University, Baltimore, MD, USA). Production and purification of the recombinant viruses were performed as described in detail previously [25], [63]. The tricistronic virus encoding for Ci-VSP and the two PH-domains will be termed Ad-PIP_2_-tool.

The cDNA of a muscarinic M_3_ receptor (M_3_-R) was amplified by RT-PCR from rat brain total RNA with the following primer pair:

5′–TTGGTGTGTTCTTCCTTGGAC-3′

5′-CAGGTAGCTTCTCTCCCGTG-3′

The M_3_-R-cDNA with stop codon was cloned into the XhoI/KpnI site of pECFP-N1.

### Cell Culture and transfection

HEK293 cells (ATCC) were cultured on glass coverslips in DMEM supplemented with 2 mM glutamine, 10% fetal bovine serum, 0.1 mg/ml streptomycin and 100 units/ml penicillin in an atmosphere of 95% air, 5% CO_2_. For transfection 2.5 µl of diluted polyethyleneimine (PEI, 1∶1000 in H_2_O) were mixed with serum free DMEM and plasmid DNA in a total volume of 200 µl and incubated for 30 min at room temperature. 800 µl serum free DMEM were added to this mixture. Cells were incubated in this solution containing 1 µg of Vector DNA for 3 h. Measurements were performed 48 h to 96 h after transfection.

### Adult rat cardiac myocytes

Wistar Kyoto rats of either sex were killed following protocols approved by the animal welfare officer of the Ruhr-University Bochum in accordance with the guidelines of the European Community (86/609/EEC). All efforts were made to minimize animal suffering. The method of enzymatic isolation of myocytes and serum-free culture conditions have been described in detail elsewhere [64]. Cells were plated at low density of about 10^3^ per dish (36 mm; µdishes^®^, Ibidi) in serum-free M199. Cells were infected about 24 h after plating. As a rule, viral titres were adjusted to yield about 50% fluorescent cells on day 3 after infection. Measurements were performed 3–5 d after infection.

### Western Blotting

Non-confluent HEK293 cells grown on 35 mm culture dishes were transfected and lysates were collected after 48 h. Samples were run in 12,5% SDS PAGE Gels and transferred to PVDF membranes (Millipore). Membranes were probed with anti GFP rabbit polyclonal antibody (ab290, Abcam, 1∶1000). The secondary antibody was anti rabbit HRP (Acris Antibodies; 1∶10000). Membranes were developped using an ECL detection kit (GE Healthcare) and analyzed in a Fuji LAS-4000 Chemiluminescent imager. Similarly, blots from protein extracts of cultured atrial myocytes (approx. 5×10^3^ cells) infected with Ad-PIP_2_-tool were generated.

### Solutions and Chemicals

For FRET and concurrent FRET and whole cell current measurements an extracellular solution of the following composition was used (mmol/L): NaCl 120, KCl 20, CaCl_2_ 2.0, MgCl_2_ 1.0, and HEPES/NaOH 10.0, pH 7.4. The solution for filling the recording pipettes contained (mmol/L): K^+^ aspartate 110, KCl 20, NaCl 10, MgCl_2_ 1.0, MgATP 2.0, EGTA 2.0, GTP 0.02, and HEPES/KOH 10.0, pH 7.4. Where indicated, GTP was replaced by GTP-γ-S (100 µM). The difference in K^+^-concentrations between intra- and extracellular solutions resulted in a Nernst potential of −50 mV.

Standard chemicals were from Merck. EGTA, HEPES, MgATP, Acetylcholine, GTP, GTP-γ-S were from Sigma.

### FRET Measurements and Imaging

FRET measurements were performed using an Axiovert 200 inverted microscope (Carl Zeiss) with a x100 oil immersion objective, a dual-emission photometric system, and a polychrome V lightsource (TILL Photonics). Illumination time was ≤50 ms at a frequency of 2–20 Hz. Excitation wavelength was set to 436±10 nm (beam splitter dichroic long pass 460 nm) and emission of single cells was recorded at 535±15 nm and 480±20 nm (beam splitter dichroic long pass 505 nm). Signals were measured by amplified photodiodes (TILL Photonics), digitized (Digidata 1322A; Axon Instruments), and stored on a personal computer using Clampex 9.0 software. FRET ratios (F_EYFP_/F_ECFP_) were corrected for direct excitation of EYFP (17% of F_EYFP’total’_) and bleed-through (74% of F_ECFP_). Wide field fluorescence images were taken using a Zeiss AxioCam MRm camera and AxioVision software.

### Current measurement

Standard patch-clamp equipment was attached to the optical setup. Whole cell GIRK channel K^+^ currents were measured as inward currents at a holding potential of −90 mV as described in detail previously (e.g. reference [31]). Application of different solutions was performed by means of a custom-made solenoid-operated flow system. All experiments were performed at ambient temperature (21–24°C).

### Statistical Analysis

All data are presented as mean±SE. Student t-test was used to compare the means between two treatment groups. P-values less than 0.05 were considered statistically significant.

## Supporting Information

Figure S1 **Schematic representation of the 2A-peptide vectors for simultaneous expression of Ci-VSP and PH-PLCδ1 fused to EYFP and ECFP respectively.** T2A and P2A denote 2A-peptide encoding sequences of *Thosea asigna* and porcine Teschovirus.
(TIF)

Figure S2 **Stable activation of GIRK-current in atrial myocyte by GTP-γ-S in the pipette filling solution.** A: Recording of membrane current as described in detail in “Materials and methods”. At the times indicated Acetylcholine (ACh, 10 µM) was superfused, resulting in stable activation of GIRK channel current. Total inward-rectifying current was assessed by superfusion of a solution containing 2 mM BaCl_2_. The rapid vertical deflections represent changes in membrane current due to voltage ramps from −120 mV to +60 mV within 500 ms applied at 0.1 s^−1^. A current-voltage relation from the ramp labeled “a”, corrected by subtraction of Ba^2+^-insensitive leak current is plotted in B.
(TIF)

## References

[1] Di Paolo G, De Camilli P (2006). Phosphoinositides in cell regulation and membrane dynamics.. Nature.

[2] Hilgemann DW, Ball R (1996). Regulation of cardiac Na^+^,Ca^2+^ exchange and K_ATP_ potassium channels by PtdIns(4,5)P_2_.. Science.

[3] Sui JL, Petit-Jacques J, Logothetis DE (1998). Activation of the atrial K_ACh_ channel by the βγ subunits of G proteins or intracellular Na^+^ ions depends on the presence of phosphatidylinositol phosphates.. Proc Natl Acad Sci USA.

[4] Hilgemann DW (2007). On the physiological roles of PtdIns(4,5)P_2_ at cardiac Na^+^- Ca^2+^ exchangers and K_ATP_ channels: a long journey from membrane biophysics into cell biology.. J Physiol.

[5] Hernandez CC, Zaika O, Tolstykh GP, Shapiro MS (2008). Regulation of neural KCNQ channels: signalling pathways, structural motifs and functional implications.. J Physiol.

[6] Suh BC, Hille B (2008). PtdIns(4,5)P_2_ is a necessary cofactor for ion channel function: how and why?. Annu Rev Biophys.

[7] Deane JA, Fruman DA (2004). Phosphoinositide 3-kinase: diverse roles in immune cell activation.. Annu Rev Immunol.

[8] Cockcroft S, Carvou N (2007). Biochemical and biological functions of class I phosphatidylinositol transfer proteins.. Biochum Biophys Acta.

[9] Halet G (2005). Imaging phosphoinositide dynamics using GFP-tagged protein domains.. Biol Cell.

[10] Szentpetery Z, Balla A, Kim YJ, Lemmon MA, Balla T (2009). Live cell imaging with protein domains capable of recognizing phosphatidylinositol 4,5-bisphosphate; a comparative study.. BMC Cell Biol.

[11] Meyer T, Wellner-Kienitz M-C, Biewald A, Bender K, Eickel A (2001). Depletion of phosphatidylinositol 2,4 bisphosphate by activaton of PLC-coupled receptors causes slow inhibition but not desensitisation of GIRK current in atrial myocytes.. J Biol Chem.

[12] Suh BC, Horowitz LF, Hirdes W, Mackie K, Hille B (2004). Regulation of KCNQ2/KCNQ3 current by G protein cycling: the kinetics of receptor-mediated signaling by G_q_.. J Gen Physiol.

[13] Cho H, Kim YA, Yoon JY, Lee D, Kim JH (2005). Low mobility of phosphatidylinositol 4,5-bisphosphate underlies receptor specificity of G_q_-mediated ion channel regulation in atrial myocytes.. Proc Natl Acad Sci U S A.

[14] Varnai P, Thyagarajan B, Rohacs T, Balla T (2006). Rapidly inducible changes in phosphatidylinositol 4,5-bisphosphate levels influence multiple regulatory functions of the lipid in intact living cells.. J Cell Biol.

[15] Suh BC, Inoue T, Meyer T, Hille B (2006). Rapid chemically induced changes of PtdIns(4,5)P_2_ gate KCNQ ion channels.. Science.

[16] Brown DA, Hughes SA, Marsh SJ, Tinker A (2007). Regulation of M(Kv7.2/7.3) channels in neurons by PtdIns(4,5)P_2_ and products of PtdIns(4,5)P_2_ hydrolysis: significance for receptor-mediated inhibition.. J Physiol.

[17] Murata Y, Iwasaki H, Sasaki M, Inaba K, Okamura Y (2005). Phosphoinositide phosphatase activity coupled to an intrinsic voltage sensor.. Nature.

[18] Iwasaki H, Murata Y, Kim Y, Hossain MI, Worby CA (2008). A voltage-sensing phosphatase, Ci-VSP, which shares sequence identity with PTEN, dephosphorylates phosphatidylinositol 4,5-bisphosphate.. Proc Natl Acad Sci U S A.

[19] Halaszovich CR, Schreiber DN, Oliver D (2009). Ci-VSP is a depolarization-activated phosphatidylinositol-4,5-bisphosphate and phosphatidylinositol-3,4,5-trisphosphate 5′-phosphatase.. J Biol Chem.

[20] Falkenburger BH, Jensen JB, Hille B (2010). Kinetics of M_1_ muscarinic receptor and G protein signaling to phospholipase C in living cells.. J Gen Physiol.

[21] Suh BC, Leal K, Hille B (2010). Modulation of High-Voltage Activated Ca^2+^ Channels by Membrane Phosphatidylinositol 4,5-Bisphosphate.. Neuron.

[22] de Felipe P, Hughes LE, Ryan MD, Brown JD (2003). Co-translational, intraribosomal cleavage of polypeptides by the foot-and-mouth disease virus 2A peptide.. J Biol Chem.

[23] de Felipe P, Luke GA, Hughes LE, Gani D, Halpin C (2006). *E unum pluribus*: multiple proteins from a self-processing polyprotein.. Trends Biotechnol.

[24] Hasegawa K, Cowan AB, Nakatsuji N, Suemori H (2007). Efficient multicistronic expression of a transgene in human embryonic stem cells.. Stem Cells.

[25] Luo J, Deng ZL, Luo X, Tang N, Song WX (2007). A protocol for rapid generation of recombinant adenoviruses using the AdEasy system.. Nat Protoc.

[26] van der Wal J, Habets R, Varnai P, Balla T, Jalink K (2001). Monitoring agonist-induced phospholipase C activation in live cells by fluorescence resonance energy transfer.. J Biol Chem.

[27] Varnai P, Rother KI, Balla T (1999). Phosphatidylinositol 3-kinase-dependent membrane association of the Bruton's tyrosine kinase pleckstrin homology domain visualized in single living cells.. J Biol Chem.

[28] Doronina VA, Wu C, de Felipe P, Sachs MS, Ryan MD (2008). Site-specific release of nascent chains from ribosomes at a sense codon.. Mol Cell Biol.

[29] Lengler J, Holzmüller H, Salmons B, Günzburg WH, Renner M (2005). FMDV-2A sequence and protein arrangement contribute to functionality of CYP2B1-reporter fusion protein.. Anal Biochem.

[30] Krapivinsky G, Krapivinsky L, Velimirovic B, Wickman K, Navarro B (1995). The cardiac inward rectifier K^+^ channel subunit, CIR, does not comprise the ATP-sensitive K^+^ channel, I_KATP_.. J Biol Chem.

[31] Bender K, Wellner-Kienitz MC, Bösche LI, Rinne A, Beckmann C (2004). Acute desensitization of GIRK current in rat atrial myocytes is related to K^+^ current flow.. J Physiol.

[32] Huang CL, Feng S, Hilgemann DW (1998). Direct activation of inward rectifier potassium channels by PtdIns(4,5)P_2_ and its stabilization by Gβγ.. Nature.

[33] Bünemann M, Brandts B, Pott L (1996). Downregulation of muscarinic M_2_ receptors linked to K^+^ current in cultured guinea-pig atrial myocytes.. J Physiol.

[34] Shui Z, Khan IA, Tsuga H, Dobrzynski H, Haga T (2002). Role of receptor kinase in long-term desensitization of the cardiac muscarinic receptor-K^+^ channel system.. Am J Physiol Heart Circ Physiol.

[35] Murata Y, Okamura Y (2007). Depolarization activates the phosphoinositide phosphatase Ci-VSP, as detected in *Xenopus* oocytes coexpressing sensors of PtdIns(4,5)P_2_.. J Physiol.

[36] Horie M, Irisawa H (1987). Rectification of muscarinic K^+^ current by magnesium ion in guinea pig atrial cells.. Am J Physiol.

[37] Yamada M, Kurachi Y (1995). Spermine gates inward-rectifying muscarinic but not ATP- sensitive K^+^ channels in rabbit atrial myocytes. Intracellular substance-mediated mechanism of inward rectification.. J Biol Chem.

[38] Cho H, Nam G-B, Lee SH, Earm YE, Ho W-K (2001). Phosphatidylinositol 4,5-bisphosphate is acting as a signal molecule in α_1_-adrenergic pathway via the modulation of acetylcholine-activated K^+^channels in mouse atrial myocytes.. J Biol Chem.

[39] Cho H, Lee D, Lee SH, Ho WK (2005). Receptor-induced depletion of phosphatidylinositol 4,5-bisphosphate inhibits inwardly rectifying K^+^ channels in a receptor-specific manner.. Proc Natl Acad Sci U S A.

[40] Sohn JW, Lim A, Lee SH, Ho WK (2007). Decrease in PtdIns(4,5)P_2_ channel interactions is the final common mechanism involved in PKC- and arachidonic acid-mediated inhibitions of GABA_B_-activated K^+^ current J Physiol.

[41] Brown SG, Thomas A, Dekker LV, Tinker A, Leaney JL (2005). PKC-δ sensitizes Kir3.1/3.2 channels to changes in membrane phospholipid levels after M_3_ receptor activation in HEK-293 cells.. Am J Physiol Cell Physiol.

[42] Keselman I, Fribourg M, Felsenfeld DP, Logothetis DE (2007). Mechanism of PLC-mediated Kir3 current inhibition.. Channels (Austin ).

[43] Mao J, Wang X, Chen F, Wang R, Rojas A (2004). Molecular basis for the inhibition of G protein-coupled inward rectifier K^+^ channels by protein kinase C.. Proc Natl Acad Sci U S A.

[44] Szymanska E, Sobota A, Czurylo E, Kwiatkowska K (2008). Expression of PI(4,5)P_2_-binding proteins lowers the PI(4,5)P_2_ level and inhibits FcγRIIA-mediated cell spreading and phagocytosis.. Eur J Immunol.

[45] Jensen JB, Lyssand JS, Hague C, Hille B (2009). Fluorescence changes reveal kinetic steps of muscarinic receptor-mediated modulation of phosphoinositides and Kv7.2/7.3 K^+^ channels.. J Gen Physiol.

[46] Falkenburger BH, Jensen JB, Hille B (2010). Kinetics of PtdIns(4,5)P_2_ metabolism and KCNQ2/3 channel regulation studied with a voltage-sensitive phosphatase in living cells.. J Gen Physiol.

[47] Luttrell LM, Hawes BE, Touhara K, Van Biesen T, Koch WJ (1995). Effect of cellular expression of pleckstrin homology domains on G_i_-coupled receptor signaling.. J Biol Chem.

[48] Gamper N, Shapiro MS (2007). Regulation of ion transport proteins by membrane phosphoinositides.. Nat Rev Neurosci.

[49] Decher N, Gonzalez T, Streit AK, Sachse FB, Renigunta V (2008). Structural determinants of Kvβ1.3-induced channel inactivation: a hairpin modulated by PtdIns(4,5)P_2_.. EMBO J.

[50] Matsushita Y, Ohya S, Suzuki Y, Itoda H, Kimura T (2009). Inhibition of Kv1.3 potassium current by phosphoinositides and stromal-derived factor-1alpha in Jurkat T cells.. Am J Physiol Cell Physiol.

[51] Ding WG, Toyoda F, Matsuura H (2004). Regulation of cardiac I_Ks_ potassium current by membrane phosphatidylinositol 4,5-bisphosphate.. J Biol Chem.

[52] Zhang H, Craciun LC, Mirshahi T, Rohacs T, Lopes CM (2003). PtdIns(4,5)P_2_ activates KCNQ channels, and its hydrolysis underlies receptor-mediated inhibition of M currents.. Neuron.

[53] Brown DA, Hughes SA, Marsh SJ, Tinker A (2007). Regulation of M(Kv7.2/7.3) channels in neurons by PtdIns(4,5)P_2_ and products of PtdIns(4,5)P_2_ hydrolysis: significance for receptor-mediated inhibition.. J Physiol.

[54] Cho H, Lee D, Lee SH, Ho WK (2005). Receptor-induced depletion of phosphatidylinositol 4,5-bisphosphate inhibits inwardly rectifying K^+^ channels in a receptor-specific manner.. Proc Natl Acad Sci U S A.

[55] Bender K, Wellner-Kienitz M-C, Pott L (2002). Transfection of a phosphatidyl-4-phosphate 5-kinase gene into rat atrial myocytes removes inhibition of GIRK current by endothelin and α-adrenergic agonists.. FEBS Lett.

[56] Delmas P, Brown DA (2005). Pathways modulating neural KCNQ/M (Kv7) potassium channels.. Nat Rev Neurosci.

[57] Hernandez CC, Falkenburger B, Shapiro MS (2009). Affinity for phosphatidylinositol 4,5-bisphosphate determines muscarinic agonist sensitivity of Kv7 K^+^ channels.. J Gen Physiol.

[58] Leaney JL, Dekker LV, Tinker A (2001). Regulation of a G protein-gated inwardly rectifying K^+^ channel by a Ca^2+^-independent protein kinase C.. J Physiol.

[59] Szymczak AL, Workman CJ, Wang Y, Vignali KM, Dilioglou S (2004). Correction of multi-gene deficiency in vivo using a single ‘self-cleaving’ 2A peptide-based retroviral vector.. Nat Biotechnol.

[60] Sommer CA, Stadtfeld M, Murphy GJ, Hochedlinger K, Kotton DN (2009). Induced pluripotent stem cell generation using a single lentiviral stem cell cassette.. Stem Cells.

[61] Tang W, Ehrlich I, Wolff SB, Michalski AM, Wölfl S (2009). Faithful expression of multiple proteins via 2A-peptide self-processing: a versatile and reliable method for manipulating brain circuits.. J Neurosci.

[62] Hossain MI, Iwasaki H, Okochi Y, Chahine M, Higashijima S, Nagayama K andOkamura Y (2008). Enzyme domain affects the movement of the voltage sensor in ascidian and zebrafish voltage-sensing phosphatases.. J Biol Chem.

[63] Bender K, Nasrollahzadeh P, Timpert M, Liu B, Pott L (2008). A role for RGS10 in β-adrenergic modulation of G protein-activated K^+^ (GIRK) channels in rat atrial myocytes.. J Physiol.

[64] Bechem M, Pott L, Rennebaum H (1983). Atrial muscle cells from hearts of adult guinea-pigs in culture: a new preparation for cardiac cellular electrophysiology.. Europ J Cell Biol.

