# Supplementary figures and images for: A Genetically Encoded Tool Kit for Manipulating and Monitoring Membrane Phosphatidylinositol 4,5-Bisphosphate in Intact Cells
Source: PLoS One. 2011 Jun 9;6(6):e20855. doi: 10.1371/journal.pone.0020855 (PMC3111442; doi:10.1371/journal.pone.0020855)

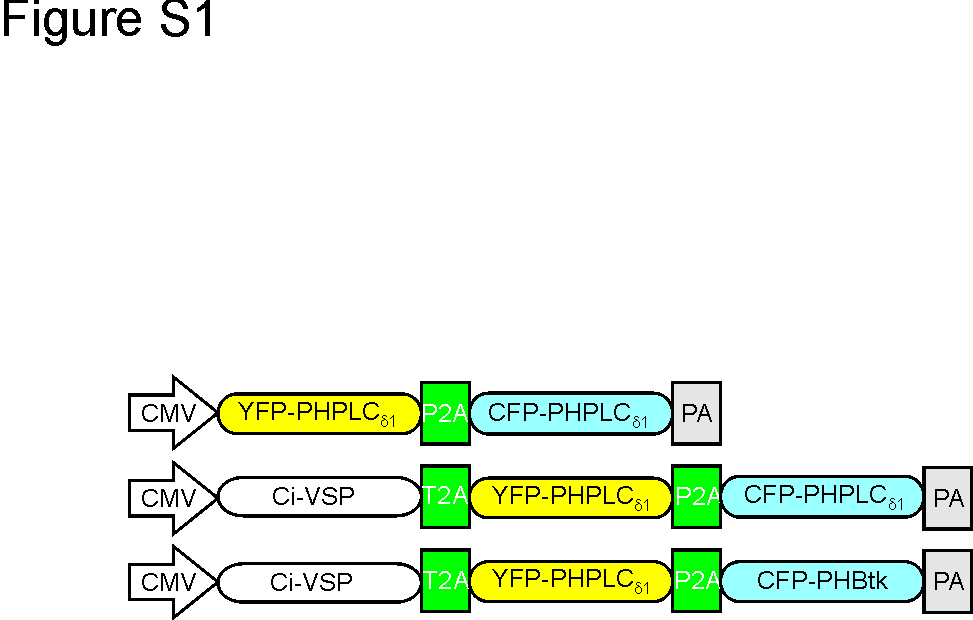

Supplement: Figure S1 — Schematic representation of the 2A-peptide vectors for simultaneous expression of Ci-VSP and PH-PLCδ1 fused to EYFP and ECFP respectively. T2A and P2A denote 2A-peptide encoding sequences of Thosea asigna and porcine Teschovirus. (TIF) [file pone.0020855.s001.tif]

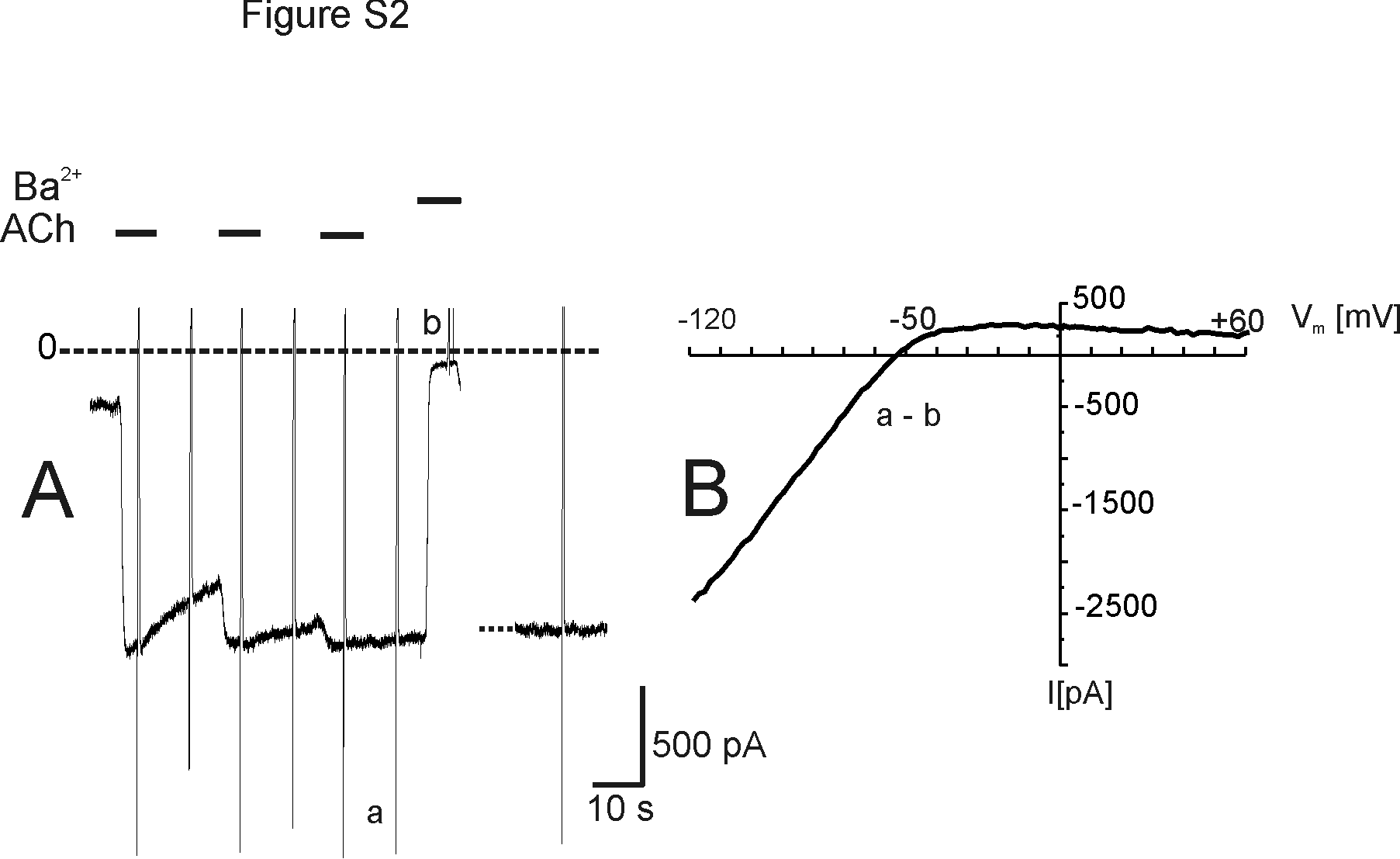

Supplement: Figure S2 — Stable activation of GIRK-current in atrial myocyte by GTP-γ-S in the pipette filling solution. A: Recording of membrane current as described in detail in “Materials and methods”. At the times indicated Acetylcholine (ACh, 10 µM) was superfused, resulting in stable activation of GIRK channel current. Total inward-rectifying current was assessed by superfusion of a solution containing 2 mM BaCl2. The rapid vertical deflections represent changes in membrane current due to voltage ramps from −120 mV to +60 mV within 500 ms applied at 0.1 s−1. A current-voltage relation from the ramp labeled “a”, corrected by subtraction of Ba2+-insensitive leak current is plotted in B. (TIF) [file pone.0020855.s002.tif]
